# The Neutral Sphingomyelinase 2 Is Required to Polarize and Sustain T Cell Receptor Signaling

**DOI:** 10.3389/fimmu.2018.00815

**Published:** 2018-04-18

**Authors:** Charlene Börtlein, Annette Draeger, Roman Schoenauer, Alexander Kuhlemann, Markus Sauer, Sibylle Schneider-Schaulies, Elita Avota

**Affiliations:** ^1^Institute for Virology and Immunobiology, University of Wuerzburg, Wuerzburg, Germany; ^2^Department of Cell Biology, Institute for Anatomy, University of Bern, Bern, Switzerland; ^3^Department of Biotechnology and Biophysics, University of Wuerzburg, Wuerzburg, Germany

**Keywords:** neutral sphingomyelinase 2, T cells, ceramides, PKCζ, the microtubule-organizing center

## Abstract

By promoting ceramide release at the cytosolic membrane leaflet, the neutral sphingomyelinase 2 (NSM) is capable of organizing receptor and signalosome segregation. Its role in T cell receptor (TCR) signaling remained so far unknown. We now show that TCR-driven NSM activation is dispensable for TCR clustering and initial phosphorylation, but of crucial importance for further signal amplification. In particular, at low doses of TCR stimulatory antibodies, NSM is required for Ca^2+^ mobilization and T cell proliferation. NSM-deficient T cells lack sustained CD3ζ and ZAP-70 phosphorylation and are unable to polarize and stabilize their microtubular system. We identified PKCζ as the key NSM downstream effector in this second wave of TCR signaling supporting dynamics of microtubule-organizing center (MTOC). Ceramide supplementation rescued PKCζ membrane recruitment and MTOC translocation in NSM-deficient cells. These findings identify the NSM as essential in TCR signaling when dynamic cytoskeletal reorganization promotes continued lateral and vertical supply of TCR signaling components: CD3ζ, Zap70, and PKCζ, and functional immune synapses are organized and stabilized *via* MTOC polarization.

## Introduction

T cell antigen recognition activates a well-defined signaling cascade involving tyrosine kinases and scaffold proteins to promote membrane proximal assembly of the T cell receptor (TCR) signalosome (1–5). This also triggers dynamic reorganization of the actin and tubulin cytoskeleton to sustain T cell activation, which relies on lateral and vertical transport of microclusters and signalosome components (6, 7). Thereby, signal is stabilized and amplified, and functional immune synapses (ISs) are organized as required for propagation and sustainment of TCR signaling (8–12). For this to occur, cytoskeleton-driven polarization of organelles, also including the microtubule-organizing center (MTOC) and the Golgi, toward the IS is a prerequisite to meet demands for enhanced vesicular trafficking of signalosome components (6). Activation of novel protein kinase C isoforms has been found to be important in this second wave of T cell activation which relies on stable anchoring of plus ends of acetylated and detyrosinated microtubuli to the peripheral SMAC, which is usually accomplished within 2–5 min (13, 14).

T cell receptor signal initiation and propagation also strongly depend on dynamic reorganization of lipid membrane domains. These are crucial for activation, segregation, or specific recruitment of signalosome components as well as cytoskeletal anchoring (10, 15, 16). This applies for instance to TCR proximal signal initiation by membrane charge-dependent conformational switching of the CD3ε cytoplasmic domain, segregation of phosphatases from active kinases or the need for displacement of cholesterol from the TCRβ chain (17–22). The lipid environment is decisive for recruitment and activity of Lck and ZAP-70 (23–25), and the importance of PIP_2_(4,5) and PIP_3_(3,4,5) enriched microdomains in PLCγ catalyzed Ca^2+^ mobilization and recruitment of PH-domain containing proteins, respectively, is well established (16, 26, 27). Furthermore, microtubuli and actin are reversibly anchored by cytoskeletal linker proteins to defined lipid areas of the IS to mediate mechanical stabilization and directional transport of subsynaptic vesicles (6, 9, 13, 28). In addition to lipid composition and turnover, lipid order and the integrity of lipid ordered domains have been found to be crucial for T cell activation (2, 16, 29–32). Those domains are highly organized and contain complex glycosphingolipids, cholesterol, and glycosylphosphatidylinositol-anchored and ace-tylated proteins. Lipid ordered membrane phases were practically defined by insolubility in non-ionic detergents. Isolation of detergent resistant membranes (DRMs) is widely used as analytic tool to isolate low-density membrane fractions for studies of lateral order of associated proteins even so results are strongly dependent on experimental conditions and seems to be artificial structures that are not present *in vivo* (33). A specific role for sphingolipids in regulation of lipid ordered domains and T cell activation, is, however, as yet ill defined.

Sphingomyelin is a major component of the plasma membrane and is a part of lipid ordered domains, and its hydrolysis by acid or neutral sphingomyelinases (ASM or NSM within the extrafacial or inner leaflet of the plasma membrane, respectively) and subsequent ceramide release was found to affect a variety of biological processes (34–38). Production of ceramides in lipid ordered domains containing sphingomyelin leads to formation of ceramide enrichment and hypothetical loss of local cholesterol (35, 39). Because of their particular biophysical properties, ceramide-enriched membrane microdomains act to compartmen-talize receptors and their proximal signalosomes and thereby regulate cellular signaling (35, 40–42). In T cells, sphingomyelin breakdown and/or ceramide accumulation can interfere with activation: depletion of extrafacial sphingomyelin caused disruption of PIP_2_ islands at the cytosolic membrane leaflet (26), ASM activity blocked phytohemagglutinin or phorbol-ester (PMA)/ionomycin stimulated Ca^2+^ mobilization (43–45), and NSM hyper-activation by measles virus abrogated co-stimulation induced actin cytoskeletal reorganization (46). Accordingly, ceramides are of low abundance in CD3-lipidomes (32) and NSM-depleted T cells were hyper-responsive to α-CD3/α-CD28-mediated co-stimulation (46). There is, however, also evidence that NSM is functionally important in TCR signaling: it is transiently activated in both α-CD3 and α-CD3/CD28 stimulated T cells, where both the enzyme and ceramides localized to the IS (46, 47).

Employing genetic depletion in primary and Jurkat T cells, we established that NSM activity is not required for initiation of TCR signaling within the first 2 min of stimulation at the level of TCR microcluster formation, CD3ζ phosphorylation, and Lck activation, but rather for TCR signal amplification needed for sustained T cell activation especially when antigen dose and co-stimulatory signals are limiting. TCR-induced sustained phosphorylation of both CD3ζ and ZAP-70 were not supported in NSM-depleted T cells, nor did these molecules efficiently polarize toward pseudo-ISs. This also applied to the MTOC and this was accompanied by α-tubulin destabilization. Importantly, essential components of the polarity complex, Cdc42 and PKCζ failed to redistribute to the IS in the absence of NSM, and this was rescued by exogenous ceramide as was MTOC recruitment. Altogether, these findings reveal that NSM activity is dispensable for initiation of TCR signaling, but is of crucial importance for its propagation and sustainment.

## Materials and Methods

### Ethics Statement

Primary human cells were obtained from the Department of Transfusion Medicine, University of Wuerzburg, and analyzed anonymously. All experiments involving human material were conducted according to the principles expressed in the Declaration of Helsinki and ethically approved by the Ethical Committee of the Medical Faculty of the University of Wuerzburg.

### Isolation of Primary Human T Cells and Generation of NSM KD Cells

Primary human PBMCs were isolated from peripheral blood obtained from healthy donors by Ficoll gradient centrifugation. CD3+ T cells were enriched (≥90%) from the PBMC fraction using nylon wool columns (Kisker Biotech GmbH). CD4+ T cells from PBMCs were negatively selected using MagniSort™ Human CD4 T Cell Enrichment Kit (Invitrogen by Thermo Fisher Scientific). Transfection of primary human T cells was done according to the manufacturer’s protocol (Lonza) using the U014 program. For silencing of NSM2, cells were nucleofected twice with an interval of 2 days with 400 pmol siRNA targeting human *SMPD3* (NSM2) (48) or, for control, a non-targeting siRNA (Sigma-Aldrich). T cells were used for sphingomyelinase assays and subsequent experiments 5 days post-transfection.

### Generation of Jurkat-ΔNSM Cells

1 × 10^7^ Jurkat T cells were transfected by electroporation (150 V) with 2 µg of both the N-SMase2 CRISPR/Cas9 KO plasmid and the N-SMase2 HDR plasmid constructs (Santa Cruz Biotechnology, Dallas, TX, USA). Cells were grown (37°C, 5% CO_2_) for up to 3 days in RPMI1640 medium (10% FBS) without antibiotics. Efficiency of the N-SMase2 CRISPR/Cas9 KO plasmid transfection was visually confirmed by GFP detection, whereas the successful co-transfection of the N-SMase2 HDR plasmid was visually confirmed by RFP detection. Doubly transfected Jurkat cells were then selected by 1 µg/ml puromycin, starting 3 days after the transfection. The selection medium was replaced every 3 days. After 3 weeks of selection, transcriptional levels of the NSM2 were assayed by qPCR.

### RT-PCR

Total RNA from 2 × 10^6^ NSM2 and control siRNA nucleofected T cells was isolated 5 days post-transfection using TRIzol Reagent (Life Technologies) following the manufacturer’s protocol. cDNA was synthesized using the First Strand cDNA Synthesis Kit (ThermoFisher Scientific) and used for PCR performed with Phusion Polymerase (ThermoFisher Scientific) and NSM2 cDNA specific PCR primers: forwards 5′ GCAGCTTCAAGTGTCTCAACAG 3′, reverse 5′ GTAGTGGGTGAACAGGGAGTGT 3′.

### Sphingomyelinase Assay

NSM activity was determined as previously described (47) with modifications. 3 × 10^6^ T cells were disrupted by freeze/thawing (methanol/dry ice) in NSM lysis buffer without detergents (20 mM Tris pH 7.4, 10 mM β-glycerophosphate, 5 µM DTT, protease inhibitors). Nuclei were removed by centrifugation for 5 min at 1,600 rpm. Post-nuclear homogenate was used directly for analysis of NSM silencing efficiency in unstimulated cells or centrifuged for 1 h at 26,000 rpm in PBS with protease inhibitors for detection of NSM activity in cellular membranes of cells pretreated with 5 µM PP2 or not (Invitrogen) for 30 min at 37°C followed by α-CD3 stimulation for different time points. Cell extracts were incubated with 1.35 mM HMU-PC in NSM lysis buffer(6-hexadecanoylamino-4-methylumbelliferyl-phosphorylcholine) (Moscerdam substrates) at 37°C for 17 h (final volume 30 µl). The ASM assay was done according to protocol provided by Moscerdam for usage of HMU-PC. Fluorescence reading was performed using excitation at 404 nm and emission at 460 nm according to the manufacturers’ protocol.

### T Cell Proliferation Assay

1 × 10^5^ T cells were pre-incubated with α-CD3- (clone UCHT-1) alone or together with CD28-specific antibody (clone CD28.2) (at indicated concentrations) (both: Beckton-Dickinson Biosciences Pharmingen) on ice for 20 min, subsequently transferred to 96-well plates pre-coated with 25 µg/ml α-mouse IgG (Dianova) (1 h at 37°C). NSM KD or CTRL T cells were stimulated for 72 h including a final 24 h labeling period ([3 H]-thymidine) (Amersham), and proliferation was analyzed using a microplate scintillation counter.

### Detection of Apoptosis

CTRL and NSM KD T cells were left unstimulated or stimulated for 24 h with α-CD3 alone or with CD3/CD28-specific Abs in combination as described above (antibody concentration: each 1 µg/ml). When indicated 400 ng/ml FasL (kindly provided by Harald Wajant, University of Würzburg) was added to α-CD3/CD28 stimulated cells for 3 h. Apoptotic cells were detected by flow cytometry using the eBioscience Annexin V Apoptosis Detection Kit (ThermoFisher Scientific).

### Detection of Membrane Order, Ca^2+^ Mobilization, TCR Signaling, and Endocytosis

#### Membrane Order

T cells were resuspended in 4 µM di-4-ANEPPDHQ (ANE, Invitrogen) in PBS for 20 min at 37°C. Fluorescence was detected at wavelengths of 570 nM (FL2 channel) and 630 nM (FL3 channel) in living cells by flow cytometry using FACS Calibur (Becton Dickinson) and analyzed by FlowJo software (TreeStar).

#### Ca^2+^-Mobilization Experiments

Following a washing step, T cells (1 × 10^6^) were loaded with 1 µM Fluo-4 as cell-permanent AM ester (Molecular Probes, Invitrogen) in Hanks balanced salt solution (without CaCl_2_, MgSO_4_, and phenol red) containing 5% FCS and 25 mM HEPES (pH 7.5) according to manufacturers’ protocol. Indicated concentrations of α-CD3 antibody (clone UCHT-1; Beckton-Dickinson Biosciences Pharmingen) crosslinked with goat α-mouse IgG (Fcγ Fragment Specific; Jackson ImmunoResearch) were added in complete Hanks medium (supplemented with 1 mM CaCl_2_) and Ca^2+^ flux over time was determined by flow cytometry.

Phosphorylated CD3ζ and Zap70 proteins in α-CD3-stimulated primary and Jurkat T cells were detected by flow cytometry using Alexa488 fluorophore conugated pCD3ζ(Y142) (clone K25-407.69) and pZap70(Y319) specific antibodies (Beckton-Dickinson Biosciences Pharmingen). Cells were stimulated by α-CD3 Ab cross-linked with goat α-mouse IgG (ratio 1:1) (final concentration: 5 µg/ml each) on ice for 15 min followed by transfer to 37°C for different time intervals. Stimulation was stopped on ice. Cells were immediately fixed (4% PFA/PBS) on ice and at room temperature (20 min each) followed by extensive washing, overnight incubation with antibody in 0.33% saponin/PBS buffer at 4°C, and analyzed by flow cytometry.

T cell receptor endocytosis was analyzed as previously described (49). 10^7^ cells/ml T cells were washed twice with cold PBS, incubated with 5 µg/ml α-CD3 antibody for 30 min on ice, shifted to 37°C, and endocytosis was stopped by incubating cells on ice and adding cold PBS prior to flow cytometry.

### Image Acquisition for *d*STORM Analyses

CTRL and Jurkat-ΔNSM cells were seeded into 8-well Poly-d-Lysin coated Lab-Teks (Lab-Tek II, Nunc; Thermo Fischer Scientific) (1.5 × 10^5^ cells/well) and adhered for 24 h at 37°C. Alexa Fluor 647 conjugated α-human CD3 antibody (UCHT1 clone, BioLegend) was pre-incubated with goat α-mouse IgG, Fcγ Fragment Specific (Jackson ImmunoResearch) for 25 min at RT (ratio 1:1, 5 µg/ml). Cells were cooled on ice for 10 min, stained with antibody-Fc solution for 30 min and left on ice for unstimulated samples or stimulated by 5 or 10 min incubation at 37°C. Cells were washed twice with ice-cold PBS and re-stained with 5 µg/ml α-CD3 antibodies for 30 min on ice. Cells were fixed (4% formaldehyde/0.2% glutaraldehyde) for 15 min at RT, washed three times with PBS and stored at 4°C until imaging. Confocal images were acquired on a LSM-700 (Zeiss) equipped with a Plan-Apochromat 63 × 1.4 oil objective. Alexa Fluor 647 fluorophores were excited with 5% transmission of a 5 mW solid-state laser at 639 nm. The emission light was detected by a photomultiplier. All images were normalized to gray values ranging from 1 to 5,000 using ImageJ (50). Super-resolution imaging of CD3 molecules was performed in switching buffer (pH 7.4) containing 100 mM β-mercaptoethylamine (Sigma-Aldrich) in PBS. *d*STORM measurements were performed using an Olympus IX-71 inverted wide-field fluorescence microscope equipped with an oil-immersion objective (60×, NA 1.45; Olympus). A 639 nm diode laser (Toptica) was used to excite Alexa Fluor 647 fluorophores. The excitation light was spectrally cleaned by a clean-up filter (Laser Clean-up filter 640/10; Chroma). Emission light was filtered by a dichroic mirror (HC560/659; Semrock) and a longpass filter (LP647; Semrock) and then projected onto an electron-multiplying CCD camera (iXon DU-897; Andor). Addition of lenses in the detection path led to a final pixel size of 134 nm. Total internal reflection fluorescence illumination was used for imaging of CD3 molecules on the basal membrane of the cells. For each *d*STORM measurement 15,000 images with an exposure time of 20 ms and an irradiation intensity of ~5 kW/cm^2^ were recorded.

### *d*STORM Image Reconstruction and Data Analysis

The software rapidSTORM 3.2 was used to localize single-molecule events in all recorded images, to generate a localization file with all localizations, and to reconstruct the *d*STORM image. Localizations containing less than 1,400 photons per frame were discarded. The spatial distribution of CD3 localizations was analyzed with custom-written Mathematica code (Mathematica, Version 11.0, Wolfram Research Inc., Champaign, IL, USA). We defined the basal membrane of each cell with all detected localizations as region of interest (ROI). For each ROI, localization clusters that represent single CD3 entities (target protein with primary antibody) or collections thereof were identified using the DBSCAN algorithm (eps = 20 nm, minPts = 3). Various cluster properties were then calculated and clustered localizations with a cluster area less than 100 nm^2^ were discarded. We compared the cluster density in each ROI and the localization density in each cluster for wild type and ΔNSM Jurkat cells.

### Cell Stimulation, DRM Isolation, and Western Blot Analysis

For DRM analyses, 2.5 × 10^7^ Jurkat or Jurkat-ΔNSM cells were left unstimulated or were stimulated in 200 µl of Hanks balanced salt solution (containing 1 mM HEPES, pH 7.5) at 37°C for 5 min with 3 µg of α-CD3 antibody (clone UCHT-1) premixed and crosslinked with 3 µg of goat α-mouse IgG. Stimulation was stopped by ice-cold Brij98 lysis buffer and lysates were subjected to sucrose gradient ultracentrifugation as previously described. For Western blot analyses, 2 × 10^6^ primary T cells or 1.5 × 10^6^ Jurkat cells were stimulated for the time intervals indicated by α-CD3 antibody crosslinked with goat α-mouse IgG (5 µg/ml each) in solution or Jurkat cells were stimulated by PMA (40 ng/ml) and ionomycin (2.5 µg/ml), lysed in 40 µl Western blot sample buffer, freezed at −80°C followed by boiling for 5 min.

pTyr were detected using mouse monoclonal antibody specific for tyrosine-phosphorylated protein species (clone 4G10, Milli-pore). p-Lck(Y505), pSrc(Y416) (rabbit mAb, D4964), pCD3ζ(Y142), pZap70(Y319), pPKCδ(Thr505), pPKCθ(Thr538), pPKCζ/λ(Thr410/403), and acetyl-α-tubulin(Lys40) (rabbit mAb, D20G3) antibodies (all from Cell Signaling) were used to detect stimulation-dependent phosphorylation of signaling molecules. Antibodies specific for CD3ζ, PKCθ (rabbit mAb, clone E1I7Y), tubulin (mouse mAb, clone DM1A) (all from Cell Signaling), and GAPDH (Santa Cruz) were used to control protein loading and normalize phosphorylation signal. PKCζ [rabbit mAb, clone EP1490(2)] from Abcam; CD3ε (rat mAb, clone CD3–12), LAT (rabbit polyclonal, FL-233), and Lck (mouse mAb, clone 73A5) antibodies from Santa Cruz were used to detect DRM associated PKCζ. Quantification of signal intensities was performed using Li-Cor software (Li-Cor Biosciences).

### Analysis of IL-2 Production

1 × 10^5^ Jurkat or Jurkat-ΔNSM cells were stimulated for 48 h with PMA and ionomycin at concentrations described for Western blot analysis. Supernatants were collected and used for IL-2 detection by ELISA (human IL-2 ELISA Ready-SET-GO, Invitrogen).

### Immunofluorescence Analysis

For pseudo-IS formation, 2 × 10^5^ NSM KD or CTRL T cells were stimulated for 15 min at 37°C in 100 µl RPMI 1640/0.5% BSA with α-CD3 (UCHT1)-coated beads (Dynabeads M-450 Tosylactivated, coated after manufacturer’s instructions, Invitrogen) at a ratio of 2:1 and captured onto a poly-l-lysine-coated slides (LabTekII, Nunc).

For Jurkat and Jurkat-ΔNSM cell polarization analysis, 1 × 10^5^ cells were pre-incubated with α-CD3 antibodies (clone UCHT-1) (1 µg/ml) on ice for 20 min, subsequently transferred to 8-well Nunc glass slides pre-coated with 25 µg/ml α-mouse IgG (Dianova) (1 h at 37°C) and stimulated for 15 min at 37°C. When indicated Jurkat cells were pretreated with 10 µM PKCζ pseudosubstrate inhibitor (PZI) (Santa Cruz) or Jurkat-ΔNSM cells with 10 µM glycogen synthase kinase 3β (GSK3β) inhibitor indirubin-3′-monoxime (IMO) (Cayman Chemical) for 30 min before cell transfer to stimulatory slides.

T cell activation was stopped by adding warm 4% PFA (in PBS) for 20 min at RT. Cells were permeabilized with 0.1% Triton-X100 for 5 min, blocked with 5% BSA, and incubated with primary antibodies specific for: TCRβ (rabbit polyclonal, H-197) (Santa Cruz), Zap70 (rabbit mAb, clone 99F2) (Cell Signaling), PKCζ (rabbit mAb, clone EP1490(2)) (Abcam), Cdc42-Alexa488 (mouse mAb, B-8) (Santa Cruz), β-tubulin (rabbit mAb, clone 9F3) (Cell Signaling), and detyrosinated α-tubulin (rabbit polyclonal) (Abcam) diluted in 1% BSA/PBS overnight at 4°C. Cells were stained with α-rabbit Alexa488-conjugated secondary antibody (Invitrogen) for 1 h at RT. F-actin was detected with 555 fluorochrom-conjugated phalloidin (cytoskeleton). To ensure that reduced fluorescence signal intensities for some signaling molecules in NSM-deficient T cells are not based on differential membrane folding, cells were loaded with fluorescent plasma membrane dye octadecyl rhodamine B chloride R18 (Sigma-Aldrich, Germany) before pseudo-synapse formation. Samples were mounted with fluorochrome G (Southern Biotech). Confocal Laser Scanning Microscopy imaging was performed using a LSM 510 Meta (Zeiss, Germany), equipped with an inverted Axiovert 200 microscope and a 40× or 63× EC Plan-Apo oil objective (numerical aperture 1.3 or 1.4, respectively) and laser lines 488 and 543. Image acquisition was performed with Zeiss LSM software 3.2 SP2. When indicated, 0.17 µm thick z-stacks were acquired and 3-dimensional reconstructed using LSM. ImageJ free software was used to analyze mean fluorescence intensities of ROIs set at pseudo-IS, plasma membrane marked by F-actin staining or inside the cell area. TCRβ, Zap70, PKCζ, and PKCθ IS interface accumulation was counted as positive if relative fluorescence increase at IS was more than 1.5 in relation to surface fluorescence intensity or intracellular accumulation. Immunofluorescence pictures were taken randomly without unconscious search of expected fluorescence patterns and all cells visible in the field were analyzed. At least 70 up to 150 cells were analyzed per condition.

### Labeling With Functionalized ω-C_16_ Ceramide

A total of 2.5 × 10^7^ Jurkat or Jurkat-ΔNSM were extensively washed and resuspended in RPMI/2% FBS containing 25 µM ω-azido-C_16_-ceramide (51), incubated overnight at 37°C and washed three times with HBSS. For click reaction, 20 µM Click-IT Alexa 488 DIBO Alkyne (Life Technologies) was added for 10 min and cells were microscopically analyzed for labeling efficiency and ceramide plasma membrane accumulation. Ceramide supplemented Jurkat cells were stimulated by α-CD3 antibody and used for lipid raft isolation, polarization on stimulatory surface followed by immunofluorescence analysis of tubulin or detection of pCD3ζ by flow cytometry.

### Statistical Analyses

Overall, data shown were acquired in at least three independent experiments and involved different individual donors when primary human T cells were used. Anderson–Darling test revealed non-normal distribution for some data sets. Therefore, non-parametric and distribution free Kolmogorov–Smirnov test was used for statistical analyses of all data sets throughout the manuscript (*p < 0.05, **p < 0.005, and ***p < 0.0001; ns, non-significant). Bars show SDs.

## Results

### NSM Activity Is Required for TCR Signaling When Antigen Dose Is Limiting

**Figure 1:**
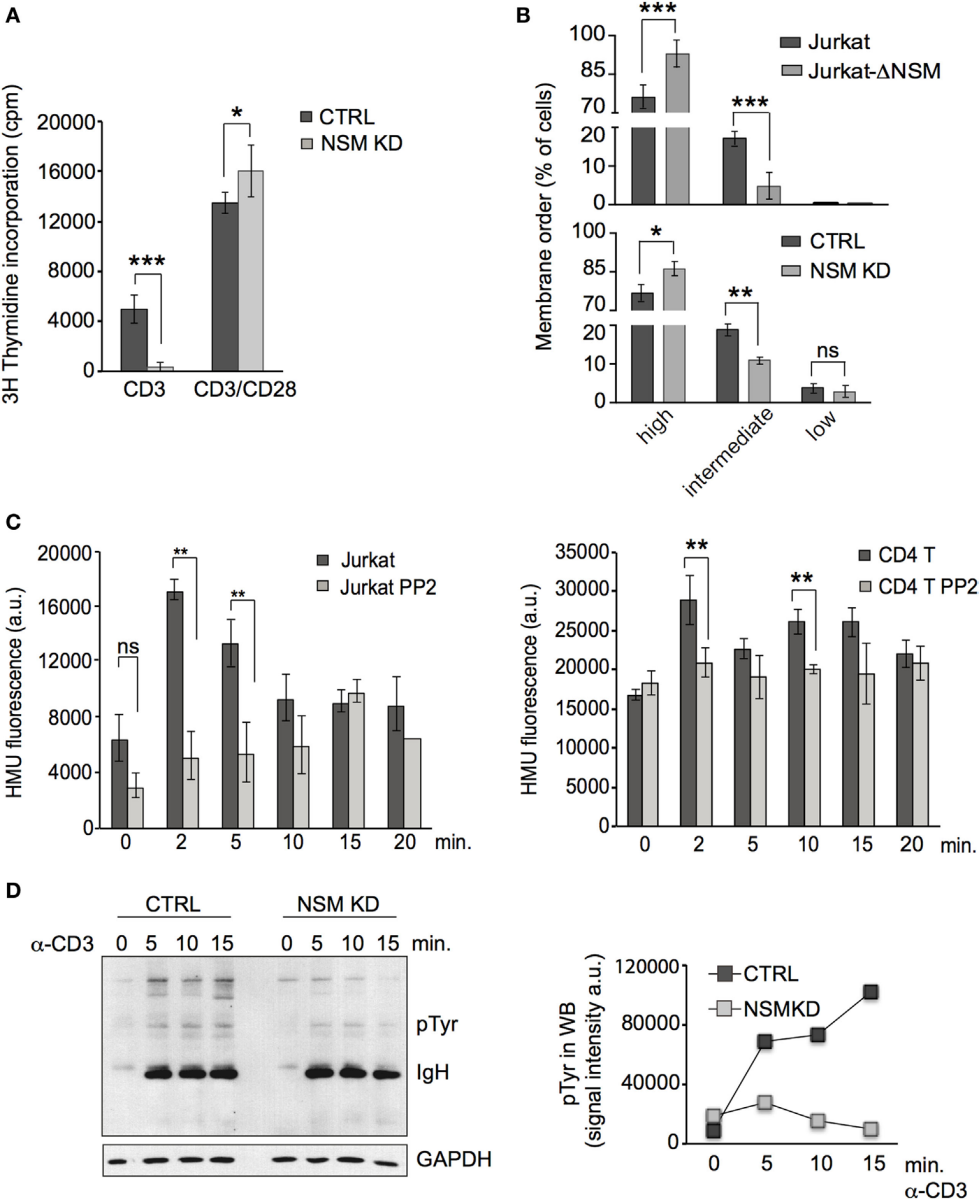
NSM activity is important in T cell receptor signaling. **(A)** CTRL (black bars) and NSM KD cells (gray bars) were stimulated by ligation of α-CD3 alone or in combination with α-CD28 (1 µg/ml each) and ^3^H-thymidine incorporation was determined after 72 h. If not stated otherwise, data were obtained and statistically analyzed from at least three independent experiments with SDs indicated. **(B)** CTRL and NSM KD or parental Jurkat and Jurkat-Δ-NSM cells were stained with ANE and fluorescence emission at 570 and 630 nm and thereby cell populations revealing high, medium, and low lipid order were distinguished. **(C)** NSM activity was determined in α-CD3-ligated Jurkat (left panel) or primary CD4+ T cells (right panel) pretreated with PP2A (each gray bars) or not (each black bars) over time. **(D)** Accumulation levels of pTyr protein species were determined in α-CD3-activated (1 µg/ml) CTRL and NSM KD cells over time (right: densitometry quantification of the lanes shown in the blot, left panel). One out of three independent experiments is shown.

To address the role of the enzyme in TCR signaling, the NSM was silenced in primary T cells by siRNA (referred to as NSM KD) or by CRIPSR-Cas9-editing in Jurkat cells (referred to as Jurkat-ΔNSM). NSM silencing in primary T cells was characterized at the level of RNA by RT-PCR (Figure S1A in Supplementary Material), and enzyme activity was reduced on average by about 50% in siRNA transfected primary T cells and by about 70% in Jurkat-ΔNSM cells (Figure S1B in Supplementary Material). Ablation of NSM activity in both systems did not affect ASM activity, cell morphology, viability, or sensitivity to induced cell death (Figures S1C–E in Supplementary Material). As described by us earlier (46), ablation of NSM activity rendered T cells highly responsive to α-CD3/CD28 co-stimulation using standard conditions (α-CD3/α-CD28, each 1 µg/ml). This was particularly pronounced 24 h after activation, however, still visible after 72 h (Figure 1A). In agreement with enhanced sensitivity to activation, a higher percentage of NSM KD and Jurkat-ΔNSM cells displayed high membrane order (Figure 1B; Figure S1F in Supplementary Material) supporting the interpretation that NSM is at least dispensable for T cell activation by co-stimulation.

Ligation of CD3 alone activated, as reported earlier (47), NSM in T cells. This peaked after 2–5 min in Jurkat cells (Figure 1C, left panel) and at 2 and 10 min in primary CD4+ T cells (Figure 1C, right panel). Pre-exposure to the Src-family kinase inhibitor PP2 prevented α-CD3-driven NSM activation thereby placing NSM activation downstream of Lck activity (Figure 1C). Expansion of T cells after CD3 ligation alone (1 µg/ml) was inefficient, but entirely abolished in NSM KD cells (Figure 1A). Accumulation of tyrosine-phosphorylated protein species was substantially reduced in both NSM KD cells and α-CD3-activated Jurkat-ΔNSM cells (Figure 1D and data not shown). These findings confirm that NSM activity supports rather than inhibits TCR signaling in the absence of co-stimulation.

**Figure 2:**
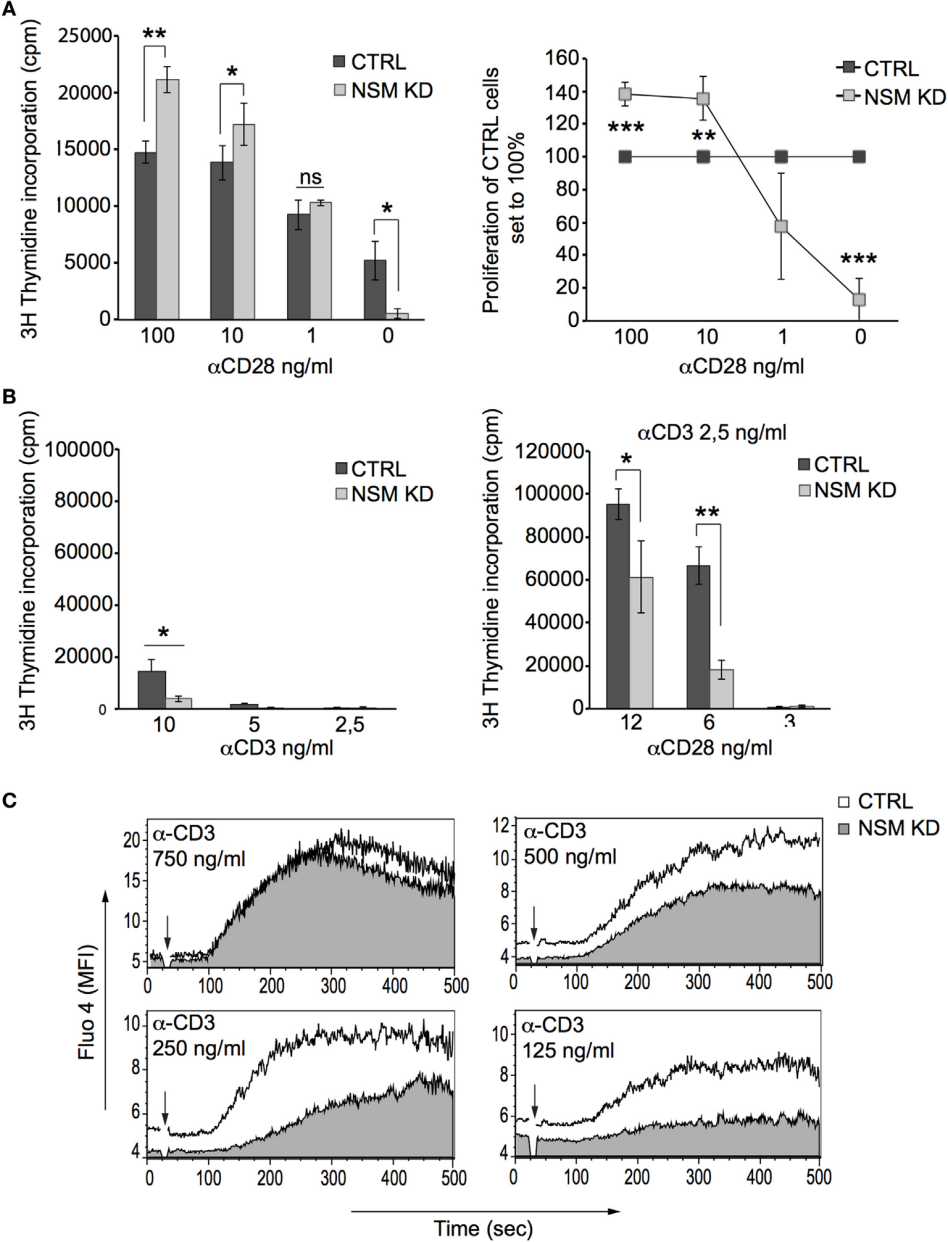
NSM is required for T cell receptor signal amplification at low antigen dose. **(A)** Proliferation of α-CD3-stimulated (0.1 µg/ml) CTRL (black bars) and NSM KD cells (gray bars) co-stimulated with decreasing concentrations of α-CD28 antibodies was determined after 72 h (left panel and proliferation of NSM KD cells normalized to that of CTRLs, right panel). **(B)** Expansion rates were determined in CTRL (black bars) and NSM KD cells (gray bars) stimulated by decreasing concentration of α-CD3 antibodies alone (left panel) or by constant α-CD3 (2.5 ng/ml) with decreasing concentrations of α-CD28 antibodies after 72 h. **(C)** Fluo-4 emission signals were recorded in CTRL (white profiles) and NSM KD cells (gray profiles) stimulated by decreasing concentrations of α-CD3 antibodies by flow cytometry over time. One representative out of three independent experiments is shown. The time point of α-CD3 addition is marked by arrow in each graph.

NSM activity is required for TCR signaling, and therefore, hyper-responsiveness of NSM KD cells should result from over-compensation of defective TCR signaling by α-CD28 co-stimulation. This could be directly shown in an experiment in which enhanced sensitivity of proliferative responses of NSM KD cells activated by a constant α-CD3 concentration (100 ng/ml) was only observed at high amounts (at least 10 ng/ml) of CD28-specific antibodies (Figure 2A). At lower concentrations of α-CD3 antibodies alone (mimicking low antigen dose), proliferative responses were lost in both NSM KD and CTRL cells (Figure 2B, left graph). Using these conditions (2.5 ng/ml α-CD3), addition of low levels of α-CD28 antibodies (6 ng/ml) still enhanced expansion of CTRL, but less so that of NSM KD cells (Figure 2B, right graph). These findings suggest that the contribution of NSM in TCR signaling could be particularly important in a physiological context where antigen doses are limiting. In support of this hypothesis, NSM KD and CTRL cells were equally efficient in mobilizing Ca^2+^ at high, but not at low concentrations of stimulatory α-CD3 antibodies (Figure 2C). Both the kinetics and magnitude of Ca^2+^ fluxing (reporting signal amplification) were substantially compromised in the absence of NSM. This strongly indicates that NSM activity in TCR signaling is of particular importance in perception and/or amplification of antigenic signals.

### NSM Activity Is Required for Sustainment Rather Than Signal Initiation of TCR Signaling

**Figure 3:**
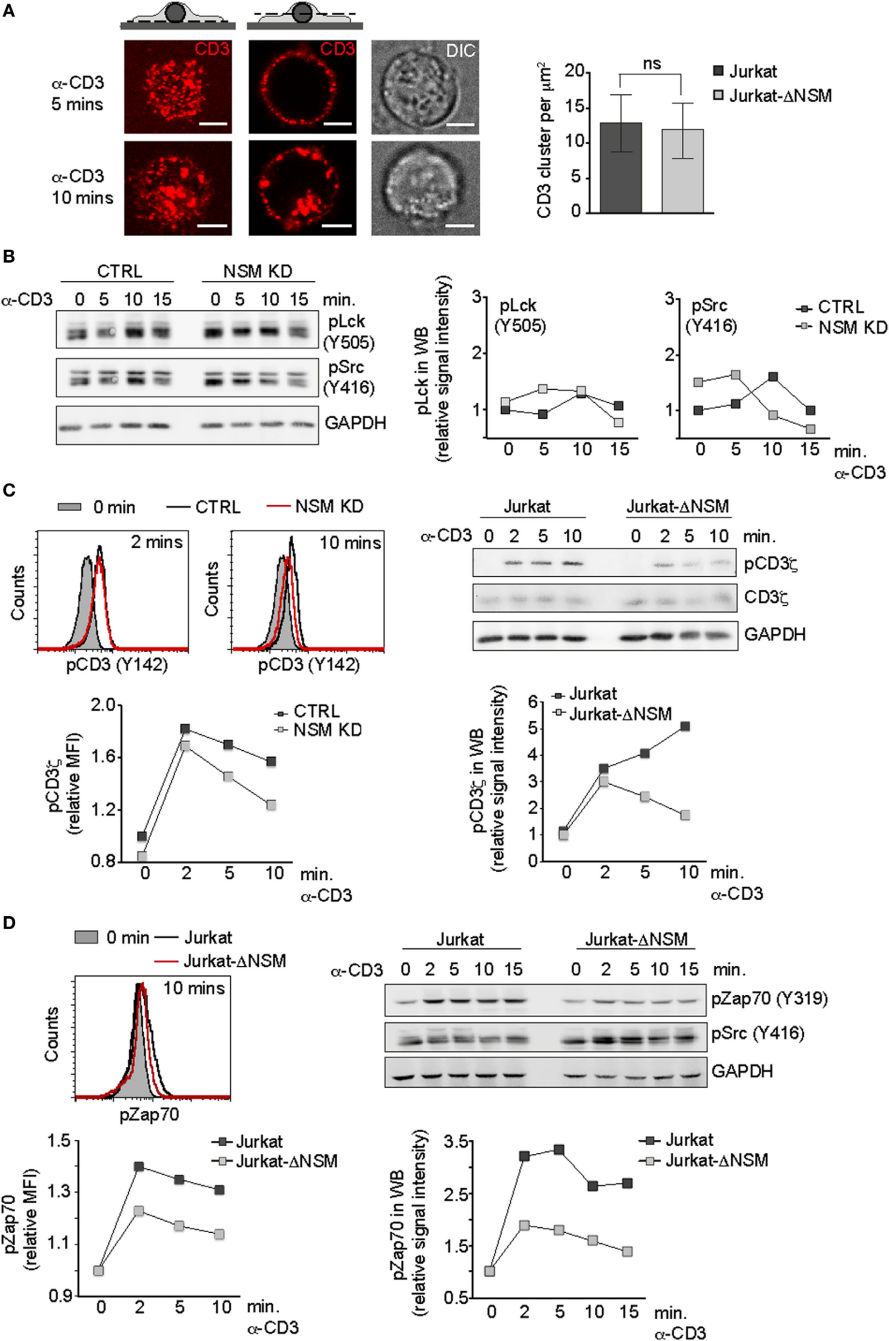
NSM activity is dispensable for initiation but not for sustainment of T cell receptor signaling. **(A)** Parental Jurkat cells were seeded on poly-l-lysin coated slides and stimulated with Alexa647-conjugated α-CD3 antibody for 5 and 10 min (IF pictures). CD3 signal localization at the basal plane is shown left (upper left pictogram and left IF pictures). CD3 endocytosis starting after 10 min of stimulation is shown in z-cut through the middle of the cell body (upper right pictogram and IF pictures in the middle). DIC cell morphology pictures are shown right. Scale bars: 5 µM. Formation of CD3-clusters was analyzed by *d*STORM at 5 min following temperature shift to 37°C and fixation. Localization of clusters was identified using DBSCAN algorithm (right graph). **(B)** Accumulation levels of pLck(Y505) and pSrc(Y416) were determined in α-CD3-activated (1 µg/ml) CTRL and NSM KD cells over time. **(C)** Phosphorylation levels of CD3ζ were determined in α-CD3-activated [as in **(B)**] CTRL and NSM KD cells (left panels, by flow cytometry) or in Jurkat and Jurkat-NSM cells (right panels, by Western blot) over time. **(D)** Phosphorylation of Zap-70 **(D)** was detected in Jurkat and Jurkat-ΔNSM by flow cytometry (left panels) and Western blot (right panels). Densitometric quantifications of the signals are shown for each of the Western blots. **(B–D)** Each one representative out of three independent experiments is shown.

To identify NSM targets in TCR signaling, we first analyzed very early steps of it. Though NSM activation in response to α-CD3 ligation is downstream of the Lck (Figure 1C), ablation of its steady state activity affects overall membrane order and therefore also possibly initiation of TCR signaling (Figure 1B). Because TCR cluster formation and receptor density therein are important in this process by favoring CD3ζ chain phosphorylation (2), these were analyzed in Jurkat-ΔNSM cells. To enable comparative monitoring of this and all subsequently described parameters, we used stimulatory α-CD3 at saturating concentrations (1 µg/ml). On poly-l-lysine seeded cells were labeled by fluorescent stimulatory α-CD3 antibodies and crosslinked by secondary antibodies on ice. To minimize confounding effects by endocytosis (which did not reveal NSM-relating differences) (Figure 3A, IF pictures left; Figure S2B in Supplementary Material) surface clusters were analyzed at the basal outer membrane 5 min after shifting cells to 37°C by *d*STORM. As revealed by quantification, formation of CD3-clusters was not affected in Jurkat-ΔNSM cells (Figure 3A). This also applied to increase in signal density within these clusters following stimulation (Figure S2A in Supplementary Material) indicating that NSM activity is dispensable for TCR signal initiation at that level.

We next addressed whether Lck activity, which is required for CD3ζ chain phosphorylation, might be affected. Overall levels of phosphorylated Lck had a tendency for slight elevation in NSM KD cells, yet this applied to both the inhibitory (pLck Y505) and the activatory (pLckY394 recognized by pSrcY416 antibody) modification and that was not significant after analysis of independent experiments (Figure 3B; Figure S2C in Supplementary Material). Moreover, the subcellular distribution of Lck proteins toward pseudo-ISs formed using α-CD3-coated beads did not reveal NSM-related differences (data not shown).

Accessibility of the Lck substrate, the CD3ζ chain, is regulated by a conformational change upon TCR stimulation. Though initially increasing in NSM KD and Jurkat-ΔNSM cells, CD3ζ chain phosphorylation levels were not maintained for longer than 2 min following stimulation (Figure 3C), which roughly corresponded to the peak of α-CD3-driven NSM activation (Figure 1C). α-CD3-stimulated phosphorylation of another Lck target, Zap-70, was weakly initiated and returned back to the level of unstimulated cells within 15 min in the absence of NSM as shown for Jurkat-ΔNSM cells (Figure 3D; Figure S2D in Supplementary Material). This indicates that NSM is not important for the initial membrane proximal wave of TCR signaling within the first 2–5 min, but rather for promoting the second wave of T cell activation required for signal amplification and sustainment.

### Polarization of Signaling Components Is Abrogated in NSM-Deficient T Cells

**Figure 4:**
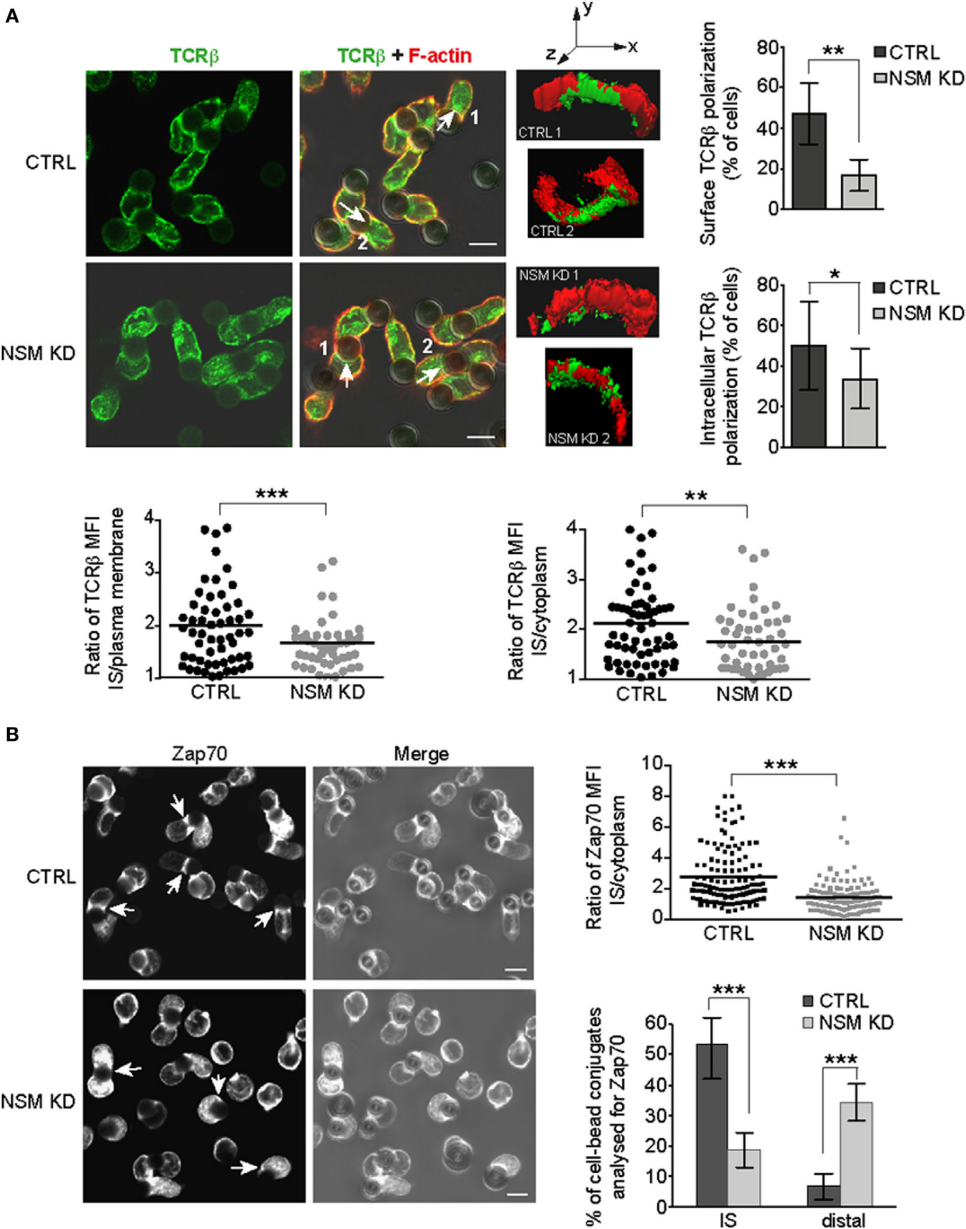
NSM activity is required for immune synapse (IS) polarization of T cell receptor (TCR) signaling components. CTRL and NSM KD cells were stimulated by α-CD3-coated beads and redistribution of TCRβ **(A)** and Zap70 **(B)** was determined after 15 min. **(A)** TCRβ was co-detected with F-actin (IF shown in left panels, right panels showing 3D reconstruction of the cell-bead IS areas indicated in the IF merge pictures by numbers and arrows). IS interface accumulation was counted as positive if relative fluorescence increase at IS of each individual cell (depicted in scatter plots) was more than 1.5 in relation to surface fluorescence intensity or intracellular accumulation. The results are shown as % of cells with positive interface accumulation of TCRβ (column bar graphs). **(B)** Zap-70 redistribution was analyzed as in **(A)** with regard to relative IS recruitment of the intracellular pool. In addition, Zap-70 re-positioning to the distal pole was observed in NSM KD cells where fluorescence intensity was more than two times higher in cell region most distal to the cell-bead interface (right bottom graph). **(A,B)** Size bars represent 5 µm.

T cell receptor signal propagation and amplification requires cytoskeletal driven polarization of signaling components and subcellular compartments toward the stimulatory interface (6, 7). We therefore comparatively analyzed redistribution of TCRβ in primary NSM KD cells forming pseudo-ISs with α-CD3-coated beads (Jurkat cells efficiently internalized stimulatory beads, and therefore, bead assays were generally performed using primary T cells). In contrast to CTRL cells, NSM KD largely failed to accumulate TCR molecules at the pseudo-IS (representatively shown by 3D reconstruction of the signals, Figure 4A). In quantitative terms, this became apparent regardless whether enrichment of the TCR signals were determined in relation to whole cell plasma membrane or cytoplasm (reporting lateral or vertical vesicular trafficking) (Figure 4A). To ensure that decreased TCR fluorescence at the pseudo-IS of NSM KD cells does not result from changes in membrane topology or different membrane folding at the cell-bead interface, CTRL, and NSM KD T cells were loaded with the plasma membrane fluorescent dye octadecyl rhodamine B chloride R18 and conjugates with beads were analyzed for fluorescence intensity at interaction interface. Broad range of R18 signal intensities was observed in different pseudo-IS, but overall fluorescence intensity distribution was similar in CTRL and NSM KD cells (Figure S3 in Supplementary Material), suggesting that TCRβ fluorescence enrichment at the interface between T cells and beads does not reflect increased membrane folding. To reveal whether lack of polarization targets also other important TCR signaling components, we analyzed redistribution of Zap-70 toward α-CD3-coated beads. As for CD3β, Zap-70 pseudo-IS accumulation was substantially impaired in NSM KD cells, with Zap-70 signals found distributed all over the cytoplasm or even at the distal pole of conjugated T cells (Figure 4B). In summary, this attributes a role of NSM in general T cell signaling machinery polarization in response to TCR activation.

### PKCs Are NSM Effectors in TCR-Driven T Cell Polarization

**Figure 5:**
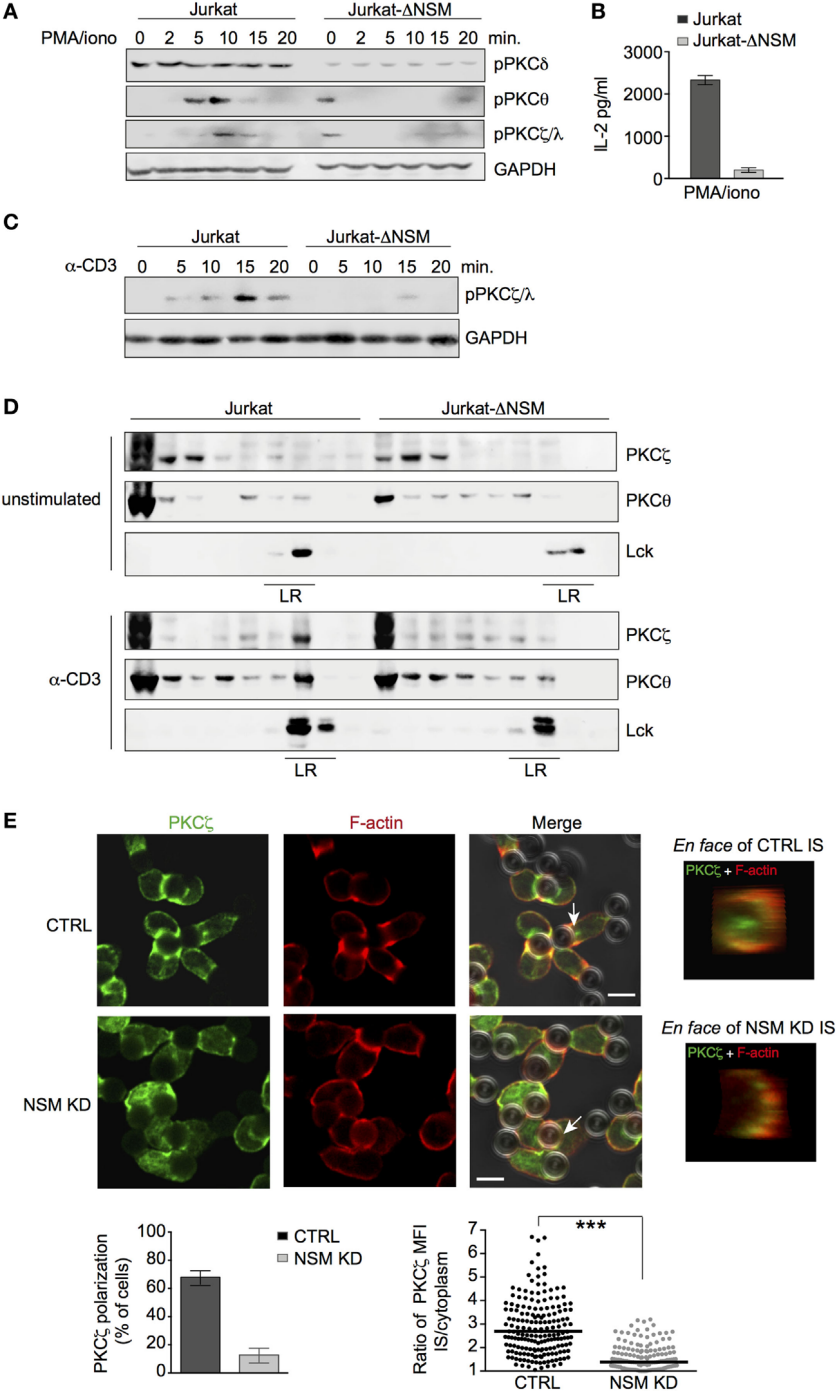
PKCζ is an NSM downstream effector. **(A,B)** Jurkat-ΔNSM and control cells were stimulated with PMA/ionomycin. Accumulation of pPKCδ, pPKCθ, and pPKCζ/λ was determined over time **(A)** and IL-2-release after 48 h **(B)**. **(C)** Accumulation of pPKCζ/λ was determined in Jurkat-ΔNSM and control cells stimulated with α-CD3 over time. **(D)** Detergent resistant membrane (DRM) domain association of PKCζ and PKCθ was determined in unstimulated (upper panels) and in 5 min α-CD3-stimulated (bottom panels) parental and Jurkat-ΔNSM cells (detection of Lck was used to identify DRM fractions). **(E)** PKCζ and F-actin were co-detected in CTRL (IF panels, upper row) or NSM KD cells (IF panels, bottom row) 15 min after stimulation with α-CD3-coated beads. The percentage of cells polarizing PKCζ (left graph) and relative polarization [immune synapse (IS)/cytoplasm] in individual cells were quantified (right graph). PKCζ IS localization was visualized by *en face* view (representative examples are shown in IF pictures on the right side). Size bars: 5 µM.

Novel (PKCθ and PKCδ) and atypical (PKCζ) PKC isoforms are important in T cell polarization (14) and both PKCδ and PKCζ are ceramide effectors (52). To reveal whether they are generally subjected to NSM-dependent regulation (independently of TCR signaling) in T cells, their activation after PMA/ionomycin stimulation was analyzed in parental and Jurkat-ΔNSM cells. Accumulation of phosphorylated PKCδ was generally low in Jurkat-ΔNSM cells and independent of stimulation (Figure 5A; Figure S4A in Supplementary Material, upper graphs). Phosphorylation of PKCθ and PKCζ/λ was stimulated by PMA/iono in unmodified Jurkat cells. By contrast, pPKCθ and pPKCζ/λ phosphorylation levels did not increase in stimulated Jurkat-ΔNSM cells after PMA/ionomycin treatment, even so their phosphorylation was detected in unstimulated Jurkat-ΔNSM cells in some experiments (Figure 5A; Figure S4A in Supplementary Material). The results indicated that these PKC isoforms require NSM for stimulation-dependent activity. This was further supported by the almost complete inability of PMA/ionomycin stimulated Jurkat-ΔNSM cells to produce IL-2, known to rely on activation of PKCs (Figure 5B). Interestingly PKCζ/λ phosphorylation was dependent on both TCR stimulation and NSM (Figure 5C; Figure S4B in Supplementary Material), whereas PCKθ phosphorylation was NSM independent (Figure S5A in Supplementary Material).

Membrane association of PKC is a prerequisite for their activation. We therefore analyzed PKC localization in α-CD3-activated Jurkat-ΔNSM cells. Both PKCθ and PKCζ partitioned into DRMs as early as 5 min following activation in control cells. This was not significantly reduced for PKCθ, but almost entirely abrogated for PKCζ in Jurkat-ΔNSM cells (Figure 5D; Figure S4C in Supplementary Material). When these analyses were extended to pseudo-IS polarization of these kinases, NSM-relating differences were not observed for PKCθ (Figure S5B in Supplementary Material). By contrast, PKCζ polarization was significantly impaired in NSM KD cells (Figure 5E). This indicated that IS recruitment of PKCζ, an organizer of cell polarity, requires NSM activity in T cells. In agreement with the PKCζ polarity complex not being adequately recruited to the IS, Cdc42, a component of this complex required for cytoskeletal reorganization, also failed to accumulate in absence of NSM (Figure S5C in Supplementary Material).

**Figure 6:**
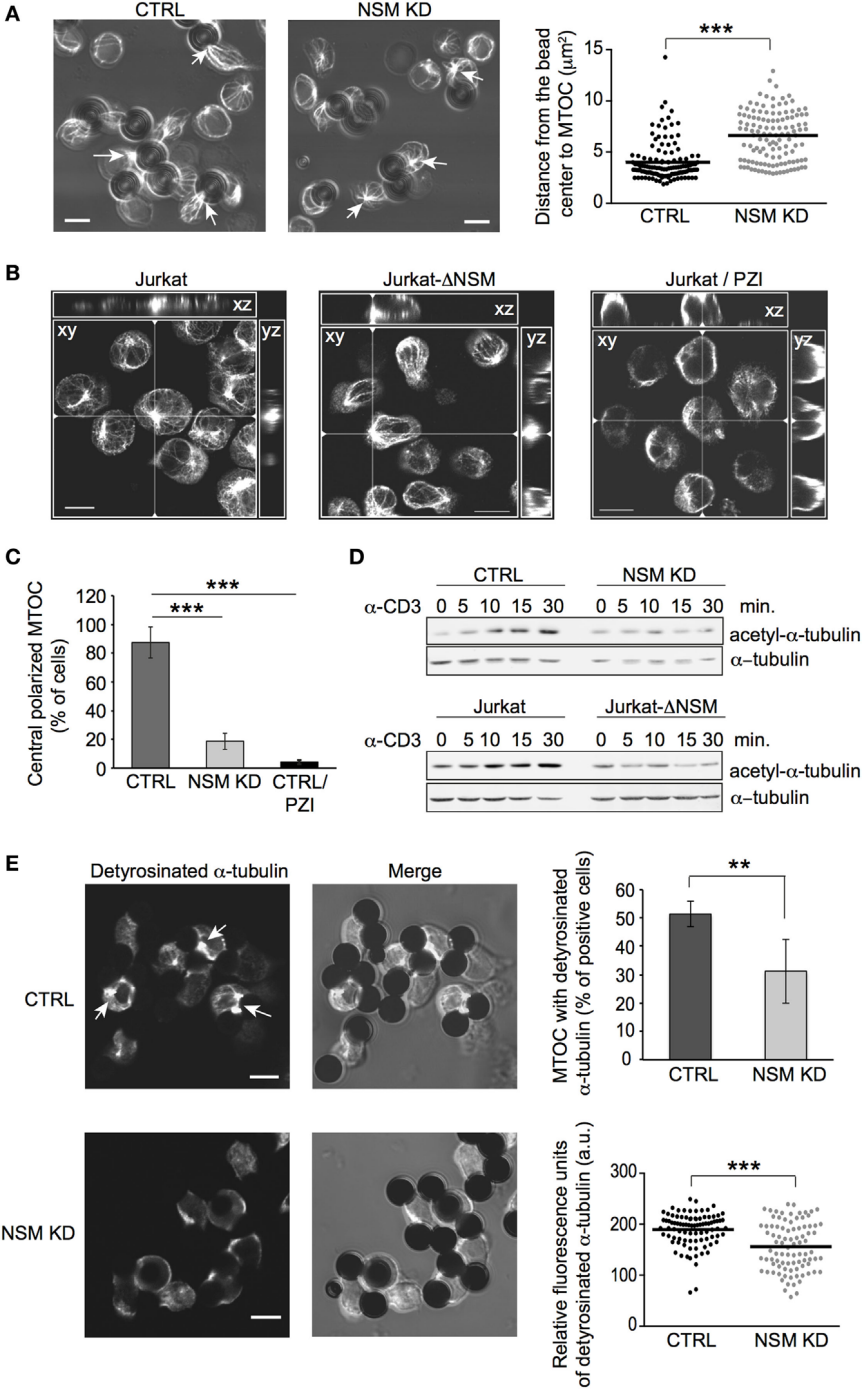
NSM is required for microtubule-organizing center (MTOC) translocation and dynamic reorganization of microtubule. **(A)** MTOC (detected by β-tubulin staining) immune synapse (IS) redistribution was determined in CTRL and NSM KD cells 15 min after stimulation with α-CD3-coated beads (examples shown in left panels). Quantification was based on the distance between bead center and MTOC, which should be less as 4 µm to consider MTOC as polarized (right panel). Arrows show examples of polarized MTOC in CTRL and non-polarized MTOC in NSM KD cells. Size bars: 5 µM. **(B,C)** Jurkat-ΔNSM and parental Jurkat cells (the latter pretreated with the PKCζ inhibitor PZI or not) were stimulated by α-CD3 on stimulatory surface coated with crosslinking antibody. MTOC structures were defined as polarized, if they were positioned in the center of cell body and recruited to the site of stimulatory surface (*xz, yz* views). Summary of evaluation of at least 70 cells in z-stacks for each condition is shown in **(C)**. Size bars: 10 µM. **(D)** Accumulation of acetyl-α-tubulin was determined in CTRL and NSM KD (upper panels) or parental and Jurkat-ΔNSM cells (bottom panels) after α-CD3-stimulation over time in Western blot. **(E)** Detyrosinated α-tubulin was detected in α-CD3-bead stimulated CTRL and NSM KD cells (IF pictures, accumulation of detyrosinated a-tubulin at IS is depicted by arrows). Percentage of cells staining positive for MTOC with detyrosinated α-tubulin is shown in upper right graph. Only cells with detyrosinated MTOC were taken for analysis of fluorescence signal intensity by ImageJ software in lower right graph. Size bars: 5 µM.

The role of PKC isoforms in MTOC polarization is established, while that of PKCζ in this process in T cells is not nor its regulation by NSM. Clearly revealing the importance of this enzyme in MTOC polarization, MTOC pseudo-IS translocation was inefficient in NSM KD cells as measured by the distance between the center of the bead and the MTOC signal (Figure 6A). Similarly, in a planar system, control, but not Jurkat-ΔNSM cells efficiently polarized the MTOC, and this was abrogated in control cells upon inhibition of PKCζ by PZI (Figures 6B,C).

Microtubule-organizing center translocation to the IS is of particular importance in sustained TCR signaling, and its relocalization relies on both PKC activation and dynamic reorganization of the microtubular network (7, 14). As established for non-T cells, NSM can regulate microtubular stability during cilia formation (53, 54). Therefore, abrogation of PKCζ IS recruitment might be accompanied by de-regulation of microtubular dynamics in NSM-depleted cells, thereby affecting both MTOC anchoring and vesicular transport. Indeed, TCR stimulation-dependent accumulation of tubulin stabilized by acetylation was strongly compromised in NSM KD and Jurkat-ΔNSM cells (Figure 6D; Figure S5D in Supplementary Material). Detyrosinated tubulin located at the MTOC initiates microtubule growth and provides mechanical forces pushing MTOC toward IS. Strikingly, almost 50% of MTOCs recorded in bead-conjugated NSM KD cells did not stain for detyrosinated tubulin (Figure 6E, upper right panel). NSM KD cells retaining detyrosinated MTOC tubulin revealed markedly reduced fluorescence signal intensities for that tubulin modification (Figure 6E, lower right panel). Altogether, these data reveal that TCR-driven IS recruitment of essential polarity complex components (PKCζ, Cdc42), its downstream effector (MTOC) and microtubular dynamics are substantially impaired in the absence of NSM.

### PKCζ Recruitment to DRM Domains and MTOC Polarization Are Rescued by Ceramide Supplementation

**Figure 7:**
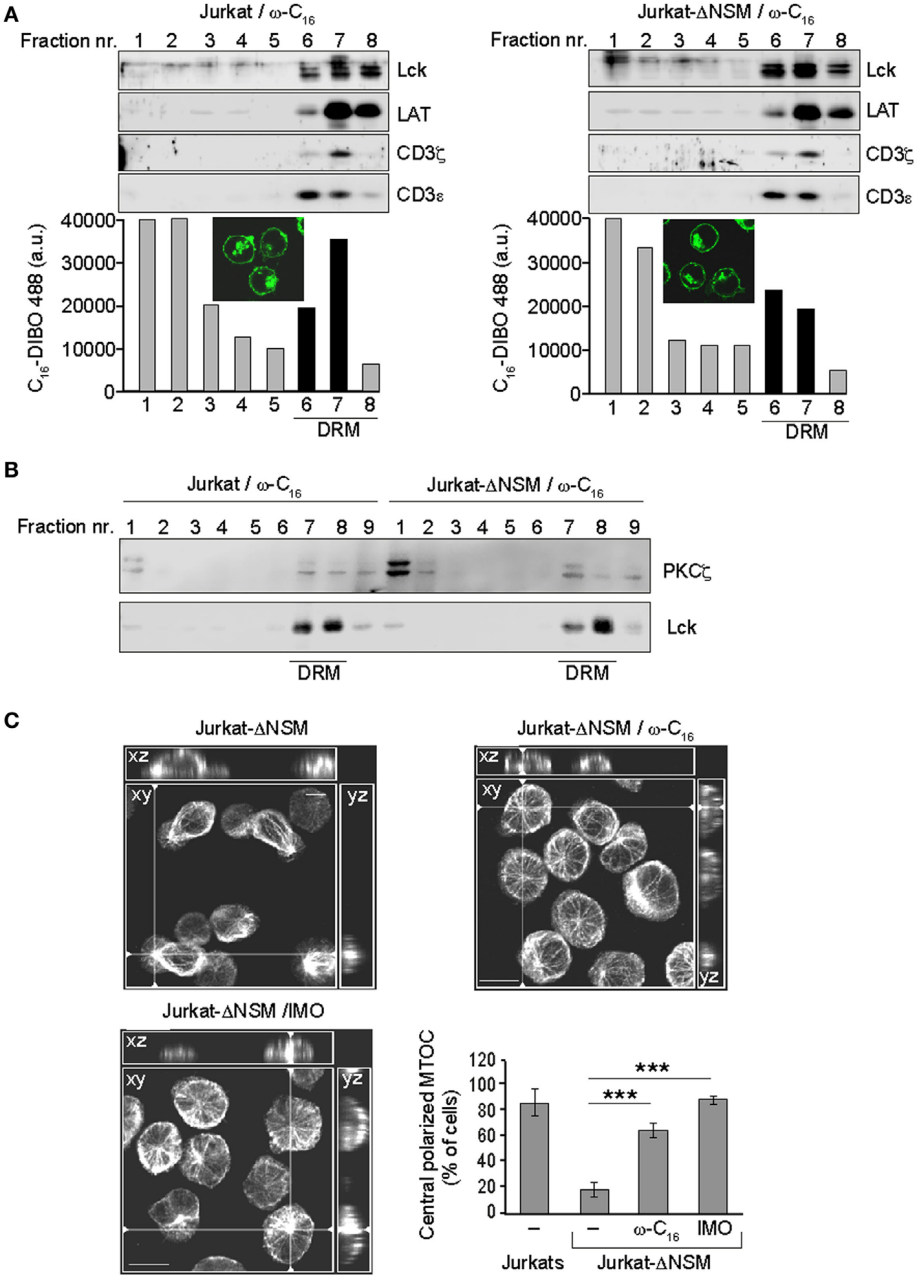
Ceramide supplementation rescues PKCζ detergent resistant membrane (DRM) association and microtubule-organizing center (MTOC) polarization in α-CD3-stimulated NSM-depleted T cells. **(A)** Parental and Jurkat-ΔNSM cells were loaded with functionalized ω-C16-ceramide, DIBO488-clicked and analyzed by IF [**(A)** insets] and subjected to floatation gradient centrifugation after which fractions containing DRM resident proteins Lck, LAT, CD3ζ, and CD3ε (serving as DRM markers) were identified by Western blot (upper panels) and accumulation of DIBO488 was measured in all fractions by fluorescence reader (bottom panels).**(B)** DRM domain association of PKCζ was determined after 5 min of α-CD3-stimulation of parental and Jurkat-ΔNSM cells pre-loaded with ω-C_16_-ceramide (detection of Lck was used to identify DRM fractions). **(C)** Jurkat-ΔNSM loaded with ω-C_16_-cermide or pretreated with the GSK3-inhibitor indirubin-3′-monoxime were α-CD3 stimulated. Graph shows the percentage of MTOC polarizing cells quantified after 15 min of stimulation as described in Figures 6B,C. Representative IF pictures for each condition are shown. Size bar: 10 µM. **(A–C)** One representative experiment out of three is shown.

Because NSM causes ceramide release and PKCζ is a ceramide effector, NSM-related defects in TCR signaling might be rescued by ceramide supplementation. To reveal whether it would redistribute to lipid rafts in parental or Jurkat-ΔNSM cells, we fed the azido-functionalized C_16_-ceramide (ω-C_16_-cer) to both cultures. Cells were DIBO-Alexa-488 clicked, α-CD3-stimulated for 5 min and imaged or used for floatation analysis. Under the conditions used, ω-C_16_-cer was efficiently incorporated and not toxic for parental and Jurkat-ΔNSM cells (as revealed by viability dye staining, data not shown). As its ω-C_6_-ceramide counterpart, ω-C_16_-cer accumulated both in the plasma membrane and an intracellular (most likely, the Golgi) compartment (51) (Figure 7A, IF insets). Upon fluorescence analysis of gradient fractions, the clicked ω-C_16_-cer also accumulated in fractions in which bona fide DRM resident proteins, such as Lck, LAT, CD3ζ, and CD3ε were detectable (Figure 7A) indicating that exogenous C_16_ indeed incorporated into TCR containing DRMs after α-CD3 stimulation. Of note, incorporation of ω-C_16_-cer into CD3, Lck, and LAT enriched fractions upon stimulation was more efficient in NSM-sufficient cells (Figure 7A, bottom graphs).

CD3ζ phosphorylation in C_16_ supplemented Jurkat-ΔNSM cells 5 minafter stimulation was similar to that in wild type Jurkat cells, while overall phosphorylation levels were reduced (Figure S6A in Supplementary Material). The reduced phosphorylation possibly resulted from the lack of tight regulation of local ceramide generation in time, normally occurring during TCR engagement. Importantly, supplementation of C_16_-ceramide rescued partitioning of PKCζ into DRM fractions in α-CD3-stimulated Jurkat-ΔNSM cells (Figures 5D and 7B; Figure S6B in Supplementary Material) and MTOC polarization toward the stimulatory surface (Figure 7C). Active GSK-3 destabilizes microtubules in immature thymocytes and its inactivation is dependent on atypical PKCs in the primary cilium (55, 56). Pretreatment with the GSK-3 inhibitor IMO rescued the ability of Jurkat-ΔNSM cells to translocate the MTOC, thereby lending further support to the importance of PKCζ as NSM and ceramide effector in stabilization of microtubuli (Figure 7C). This shows that TCR-dependent NSM activation promotes spatiotemporal formation of ceramide-enriched domains essential for T cell polarization, signal sustainment and amplification, and this is especially important when antigen dose or co-stimulation efficiencies are limiting.

## Discussion

With its potential to modify primarily the cytosolic leaflet of the plasma membrane, the NSM bears the potential to organize microdomains and thereby, formation of receptor-proximal signaling clusters (57). Pointing to its importance in T cells activation, its enzymatic activity is transiently induced by TCR ligation in the presence and absence of co-stimulation (46, 47). We now established that the NSM functions to organize sustained T cell activation which is driven by cytoskeletal-dependent polarization of signaling components toward the nascent IS, and thereby allows for signal stabilization, amplification, and sustainment. We further show that recruitment and activation of atypical PKCζ is crucial in this process.

NSM-depleted T cells were found hyper-responsive to co-stimulation, which suggested a role of the enzyme in regulating threshold activities (46). Our refined study confirmed NSM as essential in TCR signaling, and that hyper-responsiveness of NSM KD cells reflected the response to optimal stimulation where neither antigen nor levels of co-stimulatory ligand were limiting. Inside-out signaling of TCR can enhance the avidity of CD28 ligand binding and license CD28 to access TCR downstream effector pathways and thereby driving and amplifying T cell activation (58, 59). Therefore, T cells lacking signaling components especially required for cytoskeletal re-arrangements in sustaining of signal (such as NSM) can be efficiently activated only when co-stimulatory signals are particularly strong. This scenario is perfectly reflected by our findings that T cells need NSM for signal amplification at low antigen doses and unsaturated CD28-support (Figure 2B). Stimulation in the absence of NSM is dependent on highly efficient CD28 ligation (Figure 2A). To avoid co-stimulation dependent hyper-responses of NSM-depleted cells and to analyze NSM-dependent TCR signaling without co-engagement of CD28, we used antibody-based stimulation approaches lacking antigen-presenting cells.

T cell receptor-mediated NSM activation was PP2A sensitive and therefore expected to occur downstream of Lck activation (Figure 1C). Because overall lipid order is increased in NSM-depleted T cells, the enzyme also contributes to the steady state lipid composition (and loss of its activity is not compensated by ASM activity) (Figure 1B; Figures S1B,C,F in Supplementary Material). The ANEP dye reports accumulation of lipid ordered domains rather than specific contributing lipids, not allowing to draw conclusions on involved sphingolipid species. NSM-dependent membrane steady state lipid modulations could well affect initiation of TCR signaling because sphingomyelin and ceramides can regulate lateral mobility and clustering of proteins as well as lipid composition in membrane domains (60, 61). Therefore, TCRβ chain cholesterol interaction, TCR nanocluster formation, or TCR proximal nanodomain net charge essentially regulating conformational changes and accessibility of CD3ε and CD3ζ chains could have been potentially subject to regulation by basal NSM activity (2, 17, 20, 21, 62). Extensive *d*STROM based analyses did, however, not substantiate a role of NSM in TCR nanocluster formation. Because we focused on TCR signal initiation and labeled CD3 antibodies were internalized at later time points (at equal efficiencies in NSM-sufficient and -deficient cells, Figure S2B in Supplementary Material), our analyses were restricted to the first 5 min after stimulation. NSM-related differences in formation of TCR microclusters with regard to number, CD3 densities, and size were not observed (Figure 3A; Figure S2A in Supplementary Material and data not shown). Surprisingly, cluster densities in all cultures did not substantially increase upon stimulation, which was reported to enhance the likelihood of CD3ζ chain phosphorylation (2). Because CD3ζ chain phosphorylation levels were initially also not affected by the absence of NSM, we considered the mechanistic insight into discrepancies on the increase in cluster density between our and the published work (2) not within the focus of this manuscript and did not follow this aspect further.

As it is PP2 sensitive, NSM activity is not required for signaling initiation. The enzyme activity detectably increased within 2 min and declined thereafter in Jurkat and was biphasic in primary T cells (Figure 1C), while the earlier study reported peak NSM activities after 15 or 30 min in Jurkat, T cell hybridoma, and murine spleen cells (47). This different kinetic may represent differential assay sensitivities or stimulation protocols. In common to both studies, pCD3ζ was unaffected within the first 2 min of T cell stimulation. While this was not further followed in the Tonnetti study (47), we found that amplification-dependent sustainment of CD3ζ and Zap-70 phosphorylation could only be fulfilled in the presence of NSM, which was instrumental in supporting organization of a polarized vesicular sorting complex.

Extracellular vesicles of endosomal origin: exosomes are important mediators of T cell responses. They can transfer biologically active miRNA from T cells to antigen-presenting cells through immunological synapse (63) and their biogenesis in the lumen of endosomes is dependent on ceramide generated by NSM2 (64). T cells increase exosome secretion upon activation. They contain Lck, c-Cbl, and activated TCR/CD3 complexes, suggesting their role as vehicles specifically delivering signaling molecules to the site of immunological synapse in polarized fashion (65). Deficient exosome production in T cells lacking NSM could be another reason for poor TCRβ, Zap70, and PKCζ polarization toward T cell and stimulatory bead synapse.

T cell receptor effectors activating NSM remain to be identified. A fraction of NSM associates with the plasma membrane also in T cells (46), and anionic lipids, particularly phosphatidylserine (PS), are known activators (66). The latter also promotes TCR signal initiation (17, 20) suggesting that co-sorting and/or close proximity with TCR microclusters may promote NSM activation. Low abundancy of ceramides within the CD3 lipidome seems to suggest that NSM does not act in close proximity to TCR microclusters (32). Potential co-segregation of NSM with the TCR within membrane microdomains would be highly interesting to look at, was, however, beyond the scope of the present study, which focused on NSM downstream effectors in TCR signaling rather than mechanism upstream NSM activation.

Our study identified the polarity complex protein PKCζ as a major effector in NSM-dependent TCR signaling. Although nearly all protein components of polarity complex of other cell types are found in T cells, its regulation and functions upon TCR engagement at the IS is poorly understood (67). A central role of PKCζ in formation of the polarity complex in epithelial cells is well documented (68, 69). This atypical PKC also regulates chemokine-induced integrin activation and polarization in effector T cells (70), and we have recently shown that SDF-1α driven physical T cell polarization on fibronectin and inflamed endothelial cells is also NSM dependent (71). The ability of PKCζ to bind ceramide and its role in cilia formation in stem cells and neural progenitors and in polarization of primitive ectodermal cells has been previously established (54, 56, 72, 73). Prevention of ceramide accumulation by pharmacologic inhibition of ceramide synthase or NSM enabled HDAC6-dependent destabilization of primary cilia indicating that NSM participates in this process *via* activation of atypical PKCs (54). In agreement with these findings, genetic ablation of NSM prevented PKCζ membrane recruitment (Figures 5D,E), thereby showing a link between TCR-dependent NSM activation and IS recruitment of central polarity complex protein PKCζ. As highly relevant for sustainment of T cell responses, depletion of NSM substantially compromised recruitment of TCR downstream effector Zap-70 toward the IS (Figure 4B). The unique role of NSM as upstream regulator in this scenario is supported by data showing that exogenous ceramides efficiently rescue PKCζ membrane recruitment and MTOC relocalization (Figures 7B,C). The ability of exogenous ceramides, which primarily insert into the outer membrane leaflet (51, 74–76) to substitute for activation of proteins and pathways relying on NSM activity has been revealed earlier (47, 54). Whether this involves ceramide flipping or accumulation of ceramides at inner leaflet due to transbilayer communication has so far not been experimentally amenable.

PKCζ is not only a ceramide but also a PS and phosphatic acid (PA)-binding and -activated protein, and these lipid species are also enriched in IS microdomains (20, 77–79). Though not formally proven in our study, NSM activity may not affect compartmentalized PS or PA accumulation there, because it does not target TCR conformational changes (relying on PS) and Lck activity (required for DGKa activation, translocation to the IS periphery and subsequent PA generation by DAG phosphorylation). Intriguingly, both PA and ceramide accumulate in the IS periphery where centripetal actin and microtubular dependent transport of microclusters is initiated and MTOC anchoring *via* microtubuli takes place (46, 77, 80). It is therefore quite possible that TCR activated NSM and subsequent ceramide release is crucial for organizing microdomains and patterning other lipids such as PA that recruit PKCζ to promote polarization and stabilization of T cell activation.

## Ethics Statement

This study (use of primary human T cells) was carried out in accordance with the recommendations of “name of guidelines, name of committee”; with written informed consent from all subjects. All subjects gave written informed consent in accordance with the Declaration of Helsinki. The protocol was approved by the “Ethical Committee Medical Faculty University of Wuerzburg.”

## Author Contributions

Conceptualization: AD, MS, SS-S, and EA; Methodology: RS, CB, AK, and EA; Formal analysis: CB, AK and EA; Experimental investigation: CB, RS, AK, and EA; Writing: EA and SS-S; Funding acquisition: MS, AD, and SS-S; Resources: AD, RS, and AK; Supervision: MS, EA, and SS-S.

## Conflict of Interest Statement

The authors declare that the research was conducted in the absence of any commercial or financial relationships that could be construed as a potential conflict of interest.
