# Supplementary material for: The Neutral Sphingomyelinase 2 Is Required to Polarize and Sustain T Cell Receptor Signaling
Source: Front Immunol. 2018 Apr 18;9:815. doi: 10.3389/fimmu.2018.00815 (PMC5915489; doi:10.3389/fimmu.2018.00815)
Supplement: Supplementary file 1 [file Data_Sheet_1.docx]

**SFig.1**

**
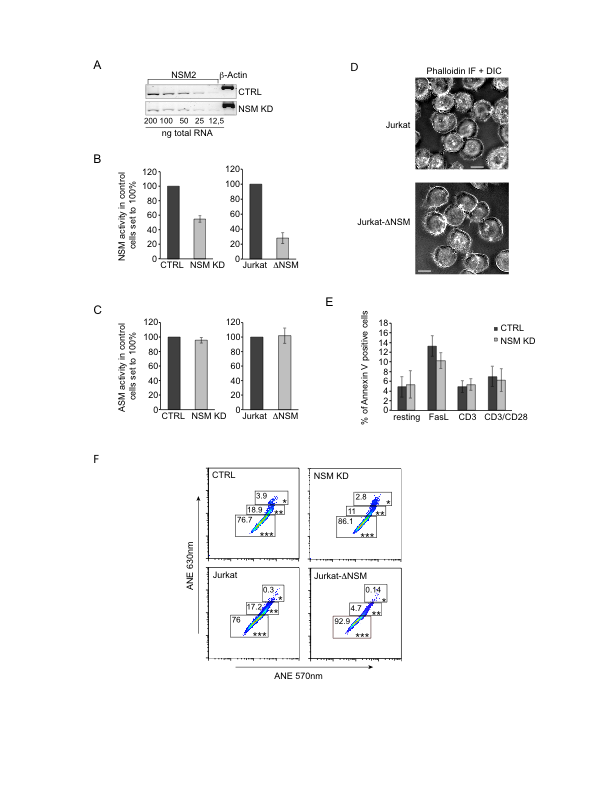
**

**SFig.2**

**SFig.3**

**SFig.4**

**SFig.5**

**SFig.6**

**Supplementary figures**

**Figure S1: Characterization of NSM2 deficient T cells.**

A. Total RNA was isolated from control CTRL and NSM KD T cells at day 5 post-transfection. Different amounts of RNA were used for RT-PCR.

B. NSM silencing efficiency in siRNA nucleofected primary human NSM KD T cells (left graph) and genetically CrispR/Cas9 modified ΔNSM Jurkat cells (right graph) was measured by NSM enzymatic activity assay. The activity values from five independent experiments were normalized against CTRL siRNA transfected T cells or parental Jurkat cells which were set to 100%.

C. NSM2 silencing effect on ASM activity was tested in NSM KD T cells (left graph) and Jurkat-ΔNSM cells (right graph). Activities of three independent measurements were normalized as in B.

D. Morphology of parental (upper panel) and ΔNSM (lower panel) Jurkat cells shown by differential interference contrast microscopy (DIC) combined with phalloidin F-actin immunofluorescence (IF) staining. Scale bar: 5 μM.

E. Analysis of AnnexinV positive CTRL and NSM KD T cells left untreated or treated with FasL, stimulated with α-CD3 alone or co-stimulated with α-CD3/CD28 for 24 hrs by flow cytometry. The graph summarizes three independent experiments.

F. CTRL and NSM KD (upper graphs) or parental Jurkat and Jurkat-Δ-NSM cells (bottom graphs) were stained with fluorescent membrane order probe ANE and fluorescence emission at 570 nm and 630 nm and cell populations revealing high ***, intermediate ** and low * lipid order (bottom, middle and upper labeled quadrants) were distinguished. One representative out of three independent experiments is shown.

**Figure S2: Differential role of NSM in CD3 clustering, endocytosis and TCR signaling.**

A. Parental Jurkat and Jurkat-ΔNSM cells were seeded on poly-L-lysin coated slides and loaded with crosslinked fluorescent α-CD3-antibodies on ice. CD3 signal density (localizations per cluster area) in clusters at the basal plane was measured following 5 min temperature shift to 37°C and fixation by *d*STORM.

B. Fluorescently labeled α-CD3 antibody was pre-bound on ice on T cells for 30 min at saturating concentrations (5 μg/ml) followed by a shift to 37°C for 10 min (Jurkat and Jurkat-Δ-NSM, left graph) or over time (CTRL and NSM KD, right graph). Surface expression of CD3 was analyzed by flow cytometry and shown as normalized mean fluorescence intensity of three independent experiments for Jurkat cells (left graph) or one representative experiment for primary T cells.

C. Summary of Western blot analyses of Lck and Src phosphorylation in α-CD3 stimulated NSM KD T cells. Densitometry analysis of Western blots of three independent experiments was done and signal intensity in NSM KD cells was compared to that in CTRL cells which was set to 1 for each time point.

D. Evaluation of Zap70 phosphorylation signals in Western blots of α-CD3 stimulated Jurkat ΔNSM cells was done as in C.

**Figure S3: NSM independent plasma membrane folding at IS with stimulatory beads.**

CTRL and NSM KD cells were incubated with α-CD3-coated beads and redistribution of fluorescence intensity of fluorescent plasma membrane dye: octadecyl rhodamine B chloride R18, was analyzed in individual cells after 15 minutes of stimulation. Results were summarized in graph. Scale bar: 5 μM.

**Figure S4: PKC activation is NSM dependent in PMA and α-CD3 stimulated T cells.**

A. Western blot densitometry analysis of PMA/ionomycin stimulated Jurkat and Jurkat ΔNSM cells. Results for PKC Western blots in Fig.5A are shown in the graphs on the left side. Graphs on the right side show the summary of all results of independent experiments for each PKC studied. Analysis of Western blots of independent experiments was done and signal intensity in Jurkat ΔNSM cells was compared to that in unmodified Jurkat cells which was set to 1 for each time point.

B. Western blot densitometry analysis of PKCζ in α-CD3 stimulated Jurkat and Jurkat ΔNSM cells. Results for Western blot in Fig.5C are shown in the graph on the left side. Graph on the right side show analysis of independent experiments done as in A.

C. PKCζ (left graph) and PKCθ DRM localization analyzed by Western blots (representatives are shown in Fig.5D). Summarized evaluation of three independent experiments was done as in A.

**Figure S5: NSM is not required for TCR dependent PKCθ activation but is essential for PKCζ/Cdc42 positive polarity complex formation and α-tubulin acetylation.**

A. CTRL and NSM KD cells were left untreated or stimulated with α-CD3 antibody for 5 and 10 min. Cell lysates were analyzed for phosphorylation of PKCθ at position Thr538 (upper panel).

B. Polarization of PKCθ in CTRL or NSM KD cells towards α-CD3 coated beads (IF pictures; arrows show PKCθ fluorescence at contact plane with the bead) was analyzed by measuring PKCθ fluorescence intensity at the pseudo-IS relative to cytoplasmic intensity in individual cells (lower graph) and results were summarized as % of cells with polarized PKCθ (polarized if ratio of MFIs at the pseudo-IS and in the cytoplasm is higher than 2) (upper graph).

C. Pseudo-ISs were formed as in B. Cdc42 and PKCζ were co-stained. IF merge shows co-localization of Cdc42 and PKCζ in CTRL cells (arrows in upper third panel) reflecting formation of polarization complex. 5 μM scale bars are shown in the right panels.

D. Densitometry analysis of acetyl-α-tubulin signal intensity in Western blots of α-CD3 stimulated NSM deficient primary T cells (NSM KD, left graphs) or NSM deficient Jurkat cells (ΔNSM, right graphs) and their NSM sufficient counterparts (accordingly CTRL and Jurkat cells). Upper graphs show analysis of representative Western blots shown in Fig.6D. Lower graphs show summary of Western blot analysis of independent experiments. Signal intensities in NSM deficient cells were compared to those in unmodified control cells set to 1 for each analyzed time point.

**Figure S6: Role of C16 ceramide supplementation in PKCζ DRM association and TCR phosphorylation.**

A. Accumulation of pCD3ζ was measured in α-CD3-stimulated parental and Jurkat-ΔNSM cells pre-loaded with ω-C_16_-ceramide or not over time by flow cytometry.

B. Summary of densitometric quantitation of PKCζ DRM association in Western blots from independent experiments for untreated and ω-C16 ceramide supplemented Jurkat and Jurkat ΔNSM cells. Quantification was done as in Fig.S4.
